# Making the Case for Proactive Strategies to Alleviate Homelessness: A Systems Approach

**DOI:** 10.3390/ijerph18020526

**Published:** 2021-01-10

**Authors:** Sara Nourazari, Kristina Lovato, Suzie S. Weng

**Affiliations:** 1 Health Care Administration Department, California State University, Long Beach, CA 90840, USA; 2 School of Social Work, California State University, Long Beach, CA 90840, USA; kristina.lovato@csulb.edu (K.L.); suzie.weng@csulb.edu (S.S.W.)

**Keywords:** homelessness prevention, systems approach, affordable housing, system dynamics

## Abstract

Homelessness is a complex public health issue that affects some of the most vulnerable populations in the United States. This study takes a holistic approach to better understand and analyze the multiple factors and their interconnections that contribute to the complexity of this problem. A systems analysis was conducted by utilizing the system dynamics method, which allows analyzing and comparing trends and behavior of system outcomes for different “what if” scenarios, interventions, and policy implications. Three leverage points were identified in this study to increase the effectiveness and efficiency of the current strategies to address homelessness: (1) increasing the number of affordable permanent housing units, (2) increasing the utilization of transitional housing units or shelters by the individuals experiencing homelessness, and (3) providing preventative services to at-risk populations before the onset of homelessness. Our results demonstrate that the most effective strategy is an amalgam of the solutions identified by these three leverage points. In addition, a policy analysis was conducted to study and estimate the effectiveness of various potential scenarios. This research provides data-driven and model-based insights into how decision-makers can utilize a systems approach to analyze new policy implications and create effective solutions to homelessness.

## 1. Introduction

Homelessness is a complex public health issue that requires a comprehensive understanding of the various factors that impact individuals’ lives. According to the United States Department of Housing and Urban Development (HUD), about 554,000 people in the country are experiencing homelessness on any given night—including 58,000 families with children [1]. Previous research studies have shown that homelessness results in negative outcomes for adults and children [2,3]. Adults experiencing homelessness have poorer physical and mental health as well as shorter life expectancies compared to the general population of adults [4]. Among children, studies reveal that they often experience high rates of school mobility and grade retention as well as lower grade and test scores than average, resulting in poor academic outcomes [5]. Research has focused on some of the barriers that individuals experiencing homelessness face in accessing social services and secure housing and have found a disconnect between individuals’ needs and services available in their local communities [6,7,8,9]. Some scholarly work [9,10] has examined interventions that address the need to increase housing and social services for individuals experiencing homelessness.

However, there is a lack of knowledge in the literature about how to systematically address poor access to permanent housing, remove barriers to service utilization, and better understand public health policy implications to further prevent homelessness. Hence, a system-level solution is warranted to consider a holistic approach instead of symptoms and event sequences [11,12]. System dynamics (SD) is one of the popular tools in systems analysis. SD uses computer simulation modeling to frame, replicate, and understand complex systems that change over time [13,14]. By simulating the actual system, its interconnected subsystems, feedback loops, and causalities, SD allows the assessment of the current state as well as the comparison of possible future behaviors of the system. When utilizing this method, researchers analyze a complex system by taking it apart to understand the structural arrangements, causalities, and their emerging effects in the system.

In this paper, we utilize system dynamics to model, simulate, and provide an assessment of the current state of homelessness in Long Beach, California (CA). This work offers a deeper understanding of the behavior that the system can produce in response to various policy implications and events. By utilizing system dynamics, we aim to examine: (1) how barriers in service utilization may affect the current and future states of homelessness and (2) whether effective policies for homelessness prevention can act as a catalyst in alleviating homelessness.

## 2. Current Strategies to Address Homelessness

Homelessness is a multifaceted issue that significantly affects individuals’ and families’ well-being and social/economic mobility. Scholars have found a disconnect between individuals’ needs and available services. For instance, May [7] found that some individuals experiencing homelessness have more difficulty matching their service needs with possible housing solutions and thus avoid accessing services altogether. Studies have also examined the outcomes of homelessness prevention programs and found mixed results. For example, Rolston and colleagues [15] found that the presence of a homelessness prevention program in New York City, known as Homebase, reduced the number of shelter entries by over 60%. However, Goodman and colleagues [16] examined the same program and concluded that the average length of a family’s shelter stay was unaffected by participation in Homebase, indicating mixed results of this particular homelessness prevention program. These results highlight the existing gaps between the current housing and public health policies, services availability, and needs of individuals experiencing homelessness.

On a macro level, cities across the United States are addressing homelessness through a variety of housing and service programs, including transitional housing, Housing First, Permanent Supportive Housing (PSH), rapid re-housing, and shelters. Over the last decade, a shift has occurred in social service delivery, placing a greater emphasis on permanent housing solutions (such as permanent supportive housing and rapid re-housing) and less emphasis on transitional housing programs. The emerging evidence-based studies demonstrate that Housing First, PSH, and rapid re-housing programs are an effective solution to homelessness in which consumers access housing faster and are more likely to remain stably housed [17]. Despite these community-wide efforts that primarily address housing needs, a recent report from HUD found that homelessness increased in CA by more than 16% from 2018 to 2019, leaving more than 151,000 Californians without permanent housing. Nearly 71% of these (about 108,000 people) are living outdoors in encampments or in vehicles, the highest percentage of any state in the nation [18]. These reports indicate that there is a gap in available services and the complexity of the underlying causes of homelessness. As such, a more nuanced and holistic approach is needed to bridge this gap.

## 3. Complexity Analysis of the Issue

Often, several factors contribute to homelessness, leading to increased complexity of this problem. For instance, at the micro level, health-related issues such as a physical or behavioral health crisis or any long-term disabling condition may place individuals at risk of homelessness [19]. A person can become chronically homeless when stable housing is too difficult to maintain without assistance due to health impairments. Experiencing abuse at home, incarceration, inadequate income, mental health issues, change in family status, and problematic substance abuse can all be among the contributing factors to homelessness [20]. These experiences affect the quality of life and overall well-being of individuals and the ability to maintain housing [21]. Additionally, households unable to afford rent experience an increased risk of homelessness [22].

At the macro level, structural contributors to homelessness also include broader sociopolitical factors and events that can trigger homelessness as a result of economic stratification [8]. For example, low-income households are typically unemployed or underemployed due to a number of factors, such as a challenging labor market. In addition, a lack of affordable housing increases the risk of homelessness. Furthermore, discrimination is a significant structural risk factor, as historically marginalized minority groups are overrepresented among the homeless population in several countries [8].

According to HUD’s annual Point-in-Time national count, 30–33% of individuals experiencing homelessness have been found to be mentally ill each year, over the past ten years [1,23]. Given that individuals who are chronically homeless may have a mental health issue as well as other medical, psychiatric, and substance use disorders, multiple systems must be involved in a coordinated effort to address these complex needs. Macro- and micro-level causes of homelessness must be examined to ensure that public health policies and initiatives will lead to sustained and stable solutions in addressing homelessness. For instance, individuals who experience chronic homelessness would need medical and behavioral health support to address their tri-morbidity, and the social services system for their basic needs such as food, shelter, clothing, and eventually employment or applying for benefits [24]. Among others, reliable transportation to relevant services, the Veterans Affairs (VA) for veterans, and homeless service providers and shelters may be needed. 

Coordinated and system-oriented approaches are necessary to provide effective solutions to homelessness. From a systems theory perspective, when systems work together, the whole is greater than the sum of the parts [25]. Systems theory allows for examination of complex systems to avoid simplistic and reductionist solutions targeting isolated parts of systems [25]. Additionally, in an interdependent system, the individual parts affect and are affected by all other parts [25]. Following a systems perspective, if the systems stop exchanging information or if the services are not integrated, then a gap in services will develop, leading to the system deterioration [26]. Furthermore, if strategies in complex systems, such as the systems that are developed to address homelessness, are only part-based and do not consider the whole system, they are likely to fail [27].

In this study, a systems perspective has been applied to model and assess the complexity and interconnections of the main factors that affect homelessness, which include: (1) permanent housing initiatives and policies for the vulnerable population experiencing homelessness, (2) the extent of temporary and transitional housing services utilization by the homeless population, and (3) factors that contribute to the risk of experiencing housing insecurity before the onset of homelessness. This work applies the system dynamics (SD) approach to examine how the limited availability of services, barriers in service utilization, and the complexity of underlying causes of homelessness may affect the current and future states of this problem, and whether integrated system-level policies can effectively reduce homelessness in communities in a sustainable manner.

## 4. Materials and Methods

It is evident that homelessness is a complex problem arising in a dynamic system and cannot be solved with static methods. Dynamic systems involve continuous change to which they respond and adapt behavior over time, translating to nonlinearities in the system behavior. Hence, alleviating homelessness warrants utilizing dynamic and comprehensive approaches such as system dynamics to obtain a deeper understanding of structural elements of the system and their emerging effects, as well as the potential system responses to policy changes.

System dynamics is a powerful system-analysis tool that utilizes computer simulation to replicate and model complex problems dynamically and illustrate system’s behavior change over time. What system dynamics does is uniquely beyond acknowledging the complexity of the problem. With SD models, we are able to represent the feedback process, time delays, and nonlinearities to determine the dynamic of the system. The development of a SD model involves two high-level stages, including building and testing a model to explain the reasons for the dynamic behavior of the system and designing and testing policies that could be integrated with the model to alleviate the problem at hand [28,29]. The purpose of using SD in this study is to test how different types of intervention strategies (mainly focusing on permanent housing and prevention-centered solutions) and their combined impact can affect the state of homelessness. This allows stakeholders to gain insight into the trends of system outcomes subject to policy changes, learn about downstream effects of new policy interventions, and set justifiable goals for sustainable courses of action.

**Figure 1:**
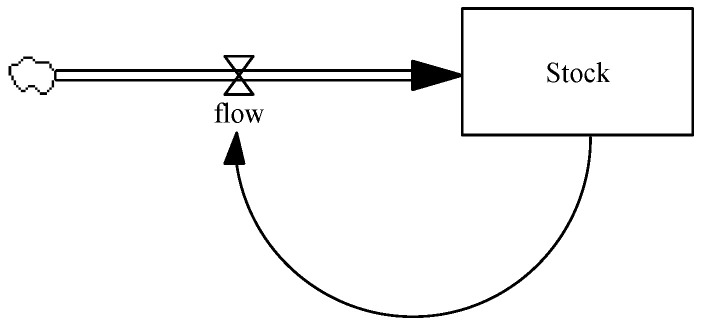
A basic stock-and-flow diagram.

System dynamics models use stocks, flows, and feedback concepts to focus our attention on implications of feedback, interconnections and causalities, delays, and accumulations over time [13,14]. In SD models, stocks (represented by rectangles) are the variables in which quantities accumulate over time. Flows (represented by arrows and valves) are the variables adding to or subtracting from the stocks. Feedback loops denote the causal relationships among the variables. Figure 1 is a depiction of a simple stock-and-flow diagram.

A basic example of a dynamic system is a bathtub, which is frequently used in introducing the SD approach. Water flowing through a faucet into the tub represents *inflow* and water leaving the tub through a drain represents *outflow*. The water accumulated in the tub represents stock that is determined by the inflow and outflow rates. In stock-and-flow maps, accumulation of stocks at a given point in time (*t*) is analogous to water level in the tub and equals the integration of net flow between the initial time (*t*_0_) and time *t*, plus the initial stock level at *t*_0_. This can be formulated as:

(1) $$Stock\left(t\right)=\int_{t_{0}}^{t}\left[inflow\left(s\right)-outflow\left(s\right)\right]ds+Stock\left(t_{0}\right),$$

Equivalently, the derivative of the stock with respect to time can be expressed as:

(2) $$\frac{d\left(Stock\right)}{dt}=inflow\left(t\right)-outflow\left(t\right),$$

where the left-hand side of the equation represents the net change rate of the stock level, and the right-hand side is the inflow less the outflow at time *t*. In the bathtub example, if the goal is to drain the water in the tub or to keep the water level as low as possible, one solution is to improve drainage (increase the outflow rate), another solution is to control the incoming water (reduce the inflow rate), and a third solution is a combination of the two aforementioned approaches. A stock-and-flow model can be developed for homelessness in a similar manner:

(3) $$\frac{d\left(homeless\;count\right)}{dt}=transitioning\;into\;homelessness\left(t\right)-transitioning\;out\;of\;homelessness\left(t\right),$$

in which *d* represents change, transitioning into homelessness is considered inflow, and transitioning out of homelessness represents outflow at time *t*. By definition and mathematically, using the stock-and-flow logic, homelessness will end in a community (homeless count = 0) when the sum of outflows from homelessness exceeds the sum of inflows into homelessness for a long enough time period. This goal can be achieved by (1) increasing the outflow rate while the inflow rate remains unchanged, (2) reducing the inflow rate while the outflow rate remains unchanged, or (3) increasing the outflow rate and reducing the inflow rate concurrently.

The SD model needs to be supported by data from the real system. Longitudinal data from homeless counts only provides information that can be used to estimate the population experiencing homelessness at a point in time (stock), which is the aggregate of all inflows and outflows of the system. However, communities with insight into a system’s inflows and outflows can develop data-driven realistic policies and objectives for permanent housing availability trends (outflow), work with policymakers on preventative measures to manage inflow (before people transition into homelessness), and develop a timeframe for eliminating homelessness. Such analysis could prove useful resource allocation and strategic planning for policymakers and social service providers.

Moreover, when developing a system dynamics model, its boundaries must be clearly defined to identify all the components that should be included in the model based on the problem definition and scope [13,14]. 

There are three different types of homelessness identified in the literature: (1) transitionally homeless, who experience relatively short and less frequent stays in temporary housing services and shelters, (2) episodically homeless, who have short but more frequent transitions into and out of homelessness, and (3) chronically homeless, who have fewer but significantly long stays [30]. As noted by HUD, individuals who are chronically homeless make up a subset of the larger homeless population [31]. Nonetheless, it is important not to overlook the needs of individuals who are marginally housed and may lack safe and adequate housing. Individuals experiencing homelessness are at increased risk for requiring emergency department services due to high rates of both unintentional and traumatic injuries from assault, poor health conditions, and high prevalence of morbidity [32].

In this study, we focus on the chronically homeless population as they are disproportionately represented in the majority (69%) of individuals living unsheltered on any given night [31]. Overall, chronic homelessness is more challenging to alleviate and significantly increases the risk of illness and premature death [33].

## 5. Results

### 5.1. Simulation Model Development and Structure

**Figure 2:**
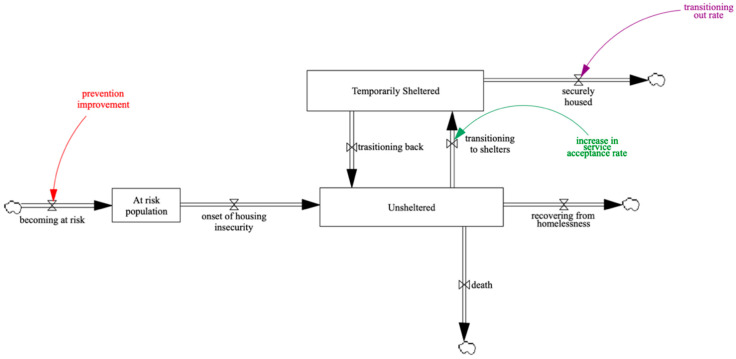
The system dynamics (SD) model, where the stocks are indicated by rectangles and flows are indicated by arrows. The cloud symbol represents the population that is not of the interest of the model and is not being tracked. The three main leverage points are indicated by three different colors.

System dynamics models heavily rely on three sources of information, including numerical data, written reports or operations manuals, and the expert opinion of key informants in the system. Data on these three main areas leads to descriptive data analysis and building the explanatory SD model, followed by policy design and evaluation [14]. In accordance with the main steps in the model development, a system dynamics model was developed that is shown in Figure 2.

The model uses publicly available data for chronic homelessness in the city of Long Beach, CA, where chronic homelessness is defined as “An unaccompanied individual or a family with a head of household with a disabling condition who (1) has been continuously homeless for a year or more, or (2) has experienced at least four separate episodes of homelessness in the past three years, where the combined episodes total a length of 12 months or more” [34]. However, the model can be modified and adjusted for other communities and cities upon the community-specific information availability.

It is worth noting that in complex problems such as homelessness, where accurate data is unavailable, numerical estimations are applied. In addition, since no model can provide “complete” accuracy, attention should be paid to the trends, insights, and relative changes rather than exact numbers. Furthermore, although SD is known to be a powerful approach in modeling interdependencies and causal loops in a system, it is not possible to capture all the details and dynamic complexities in a single SD model. For example, this study only focuses on chronic homelessness, however, there are many individuals and families who are experiencing homelessness but are not considered in the census or point-in-time count data. Those who may be couch surfing with family and friends, doubling up with strangers, or those who relocate too frequently are experiencing housing insecurity but do not appear in the data and analysis of the system state.

**Figure 3:**
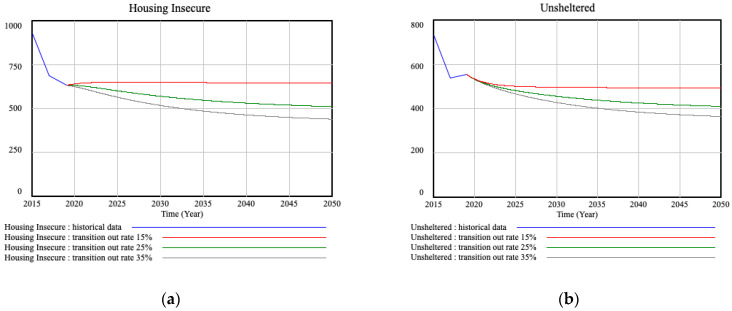
The simulation results of the model show the state of the system for the transitioning out rates of 15%, 25%, and 35%, assuming that the preventative services and temporary housing utilization rates remain unchanged: (**a**) Housing Insecure graph displays the total population at temporary housing units plus those unsheltered. (**b**) Unsheltered graph depicts the homeless count for the population not utilizing temporary housing and shelter services.

In the first stage of this study, we examined the impact of increasing the annual availably rate of the permanent housing units. This is the rate at which people in temporary housing facilities and shelters are transitioned to permanent housing units (transitioning out rate). This is analogous to increasing the outflow rate in the bathtub model. Annual transitioning out rates of 15%, 25%, and 35% were analyzed, assuming that the preventative services and temporary housing utilization rates remain unchanged. Figure 3 displays the result of this analysis.

As expected, the homeless count drops incrementally by increasing the transitioning out rates driven by different housing policies and initiatives. However, the system fails to reach 0 homeless count over the simulation time window of 30 years even with a 35% annual rate of providing affordable permanent housing. Additionally, due to the constant demand for permanent housing assistance, the system is at capacity, leading to a strained and overburdened state.

### 5.2. Policy Experiment

As policy interventions testing is implemented by means of flows in system dynamics modeling, in order to conduct a comprehensive policy experiment for the developed model, we studied how increased permanent housing availability, improvement in prevention before the onset of homelessness, and expanded utilization of temporary housing and shelters can affect the current state of homelessness in the model. In order to assess the system performance in response to different policy changes, a performance measure was developed. Time to achieve 30% drop in homeless count was applied as the performance measure of the simulation model. Table 1 summarizes the results of this analysis for various scenarios. P1 represents the policies for increasing permanent housing availability, P2 represents the policies for improving service acceptance rate for transitional and temporary housing units, and P3 represents the policies for enhancing preventative services.

The results demonstrate the notable impact of improvement in prevention and public health policies on effectiveness of housing policies and initiatives as it translates to reduced and managed demand for both temporary and permanent housing units. Combined with increased utilization of the temporary housing assistance and the current affordable housing policies, the system may achieve further improved outcomes. For instance, the system achieves a 30% drop in homeless count within 16 years with 5% improvement in prevention initiatives, 5% increase in temporary housing assistance utilization, and transitioning out rate of 15%. In a different scenario, if the preventative initiatives and utilization of the temporary housing assistance improve by 10% each, while transitioning out rate is at only 15%, the system reaches a 30% drop in homeless count in 10 years. However, when the prevention initiatives remain unchanged and at their current state with no improvement, the system can never reach the 30% drop in the homeless count even by increasing the availability of affordable housing units from 5% to 15%. Moreover, in another scenario, when the improvement for prevention-focused policies and the transitioning out rate are 5% and 15% respectively, the 10% increase of the utilization rate of transitional housing only improves the time to achieve a 30% drop in homeless count by 1 year compared to the case with a 5% increase of the utilization rate of transitional housing. It is worth noting that by undertaking more aggressive prevention initiatives, the goals for alleviating homelessness can be achieved more effectively and efficiently in a much shorter time span.

Although system dynamics does not remove the complexity of a problem, it helps develop a detailed map of the system with all the interconnections and identify the high leverage points in the system. The high leverage points of a system are defined as the ability to fundamentally improve the performance of a system, while simultaneously reducing the likelihood of negative unintended consequences. System dynamics accomplishes this by providing a simulation model to analyze policy implications and predict the system behavior in a data-driven quantifiable fashion.

Three leverage points were identified in the system based on the simulation analysis in this study. The first one is providing permanent housing units for vulnerable populations experiencing housing insecurity. This leverage point remains as the most effective and critical solution to homelessness; however, its impact could be minimal or even futile in an overburdened system with increasing demand patterns.

The second leverage point is providing preventative services to at-risk populations before the onset of homelessness. This could range from affordable or state-funded mental and physical health services, as well as rehabilitation centers for substance abuse. Providing such services could prevent or significantly delay the onset of homelessness for the vulnerable population. In addition, there are several policies that could be put in place to help an individual who loses employment to find a new job before the onset of housing insecurity due to delayed rent or mortgage payment. The emphasis of this leverage point is solely on proactive strategies to prevent transitioning into homelessness or to delay the onset of it. This could potentially lead to a balanced supply–demand system, where demand does not follow an increasing pattern and hence, meeting the demand for permanent housing for the in-need individuals could be an achievable goal. With improved public health and reduced demand, the housing policies and initiatives will be more effective in housing the homeless population. In the bathtub example, this leverage point is analogous to decreasing the inflow rate. It is worth noting that the prevention policies in this study do not consider a specific area and the emphasis is on the dynamic of prevention rather than a particular focus.

The third leverage point identified in this study is the utilization of available shelters and temporary housing services by the population experiencing housing insecurity. The Long Beach Continuum of Care identifies three paths to permanent housing, of which two reflect utilization of emergency shelters and transitional housing [20]. Availability of various housing resources allow for effective placement of people with different needs in stable housing [34]. In this model, we assume short-term or medium-term housing options as transitional stages that are designed to support moving individuals to permanent housing. The model, however, can be modified to eliminate this step. In the bathtub example, this concept is analogous to a tub where the outflow pipe is not directly connected to the tub but instead, it is connected to a tank within the tub. Only the portion of the inflow that enters the tank can exit the tub. In this case, if the flow rate from the inflow to the tank is low (analogous to individuals experiencing homelessness not utilizing transitional and temporary housing units), increasing the outflow rate (analogous to developing a larger number of permanent housing units) will not, by itself, have a considerable impact on the bathtub water level (analogous to homeless count). This leverage point in the system is worth analyzing because it is known that individuals who experience homelessness encounter barriers in accessing services and may not fully utilize resources in the transitional and temporary housing units aimed at assisting in the transition to a secure and permanent housing state [35]. Based on the 2020 point-in-time homeless count released in June 2020, the city of Long Beach, CA, experienced a 7% increase in its homeless population compared to 2019. Meanwhile, the number of people living unsheltered increased by 24% as the number of those in shelters and other temporary living situations dropped by 27% [36]. The 2020 point-in-time count indicated that of the 2034 total homeless population in Long Beach, 1582 (78%) were unsheltered. However, this rate was higher (88%) for the adults experiencing chronic homelessness [36]. There are several factors that may attribute to low utilization of shelters and transitional housing. One factor is the limited housing options for individuals and families experiencing homelessness. For instance, Long Beach has only 257 beds in emergency shelters, 339 beds in rapid rehousing, 337 beds in transitional housing, and 1469 beds in permanent housing units [20]. Another driver of the low utilization rate noted by Long Beach officials is the limited availability of crisis shelter funds for motel vouchers [36]. Yet another contributing factor to low utilization may be that individuals are unwilling or unable to take advantage of available services. In 2019, among the 1894 individuals experiencing homelessness in Long Beach, 60% of survey respondents indicated that they have been outreached for services but only 31% indicated service acceptance from the outreach [34]. However, during the 2020 point-in-time count in Long Beach, only 39% of respondents reported that they had been outreached for services, which could be due to the COVID-19 pandemic [36,37]. The reasons for not accepting services are unclear and warrants further investigation. Overcrowding, unsanitary conditions, sobriety rules that exclude individuals who use substances, and strict policies around bringing animals into facilities are among the possible reasons for not being sheltered [35]. For example, as many as 25% of individuals experiencing homelessness have pets and this was found to be associated with decreased usage of housing services [38].

Identifying and addressing these barriers can increase the utilization of the intermediate services, which at the system-level can effectively accelerate the transition to permanent housing and reduce homelessness.

## 6. Discussion

Homelessness is a complex issue for many communities and requires adaptive and system-level approaches to intervention and measurement. Maintaining adequate income and secure housing can be extremely challenging for many households. Managing outflow through housing policies and approaches such as Housing First (HF) has been the main focus of the current solutions to homelessness in major urban cities and communities of the United States. Unfortunately, these initiatives have not sustainably reduced homelessness in many communities. However, if combined with preventative strategies oriented around public health to avert the transition of vulnerable populations into homelessness and support at-risk populations, it can lead to significant results in alleviating homelessness. New policies for affordable and accessible housing, discharge planning from incarceration, subsidized mental health support, domestic violence protection services, and subsidized preventative healthcare plans are some examples of targeted preventative initiatives that can decrease the rate of transitioning from an “at-risk of homelessness” state into homelessness. Although the causal relationship between the prevention-oriented approaches and homelessness is intuitively understood, there is still a lack of sufficient research on the depth and breadth of downstream impacts of the prevention initiatives to address homelessness. This work is a call to action for further research to study the effectiveness and efficiency of specific prevention-centered policies from a systems perspective that takes into account the structural causal loops and interconnections within the system.

Providing affordable and accessible housing remains the main focus of the solutions to end homelessness, however, our results demonstrate that if it is accompanied with improvements in prevention-centered policies, the outcome could be substantial. Strategies with increased rates of transitioning the population residing at temporary housing units and shelter into permanent housing units can have a significant impact on the state of homelessness only if combined with strong prevention initiatives. Furthermore, improving the process of timely identification and transitioning of the housing insecure population to temporary housing can increase the efficiency of these policies for permanently transitioning homeless individuals out of homelessness.

Canada, Australia, and the United Kingdom are among some of the countries that have instituted wide-scale preventative efforts to reduce homelessness. For example, Canada is at the beginning stages of the move towards a stronger focus on prevention, aided by a social innovation agenda to identify, design, test, and evaluate preventative interventions to determine the most effective approaches [39]. In Australia, Spinney et al. found that homelessness can be prevented despite its complexity by shifting away from a focus on crisis intervention services and instead implementing rapid rehousing, and more tailored and appropriate housing options [40]. Meanwhile, Scotland has been leading the way with innovative approaches to implement preventative policies by using a five-category prevention typology (Universal, Targeted, Crisis, Emergency, Recovery) focused on emergency prevention—ensuring that people experiencing homelessness have a right to temporary housing. More recently, these efforts have expanded to crisis prevention, whereby people at risk of homelessness are assisted to remain or secure alternative housing within 56 days [41]. Prevention and early intervention programs are emerging globally, shifting away from crisis-oriented services and turning more towards rapid rehousing programs.

The findings in this work contribute to several implications for public health policies. Similar to results from Goodman and colleagues [16], who found that there is a gap between various services availability and needs of individuals experiencing homelessness, findings from this study can help us better understand how different policies and strategies in the real system would affect the at-risk population and individuals experiencing homelessness. For example, in Los Angeles County, Measure H and Measure HHH were passed in 2017, to provide funding for an increase in preventative services, programming, and housing for individuals experiencing homelessness. Measure H, the “Los Angeles County Plan to Prevent and Combat Homelessness”, generates funds for the specific purposes of funding homeless services, such as prevention, outreach, and short-term housing, and has increased the number of emergency beds and expanded outreach efforts. Proposition HHH allows for the building of up to 10,000 units of housing [42]. These new measures aim at providing effective outreach and intervention efforts. However, a significant amount of holistic and coordinated efforts is needed between systems such as non-profit organizations, providers, faith-based institutions, community members, housing unit owners, and local policymakers to achieve a sustainable solution for homelessness. The system dynamics method provides a practical way to help policymakers gain insights into the dynamic of homelessness. Due to its holistic nature and utilization of computer simulation, system dynamics has the potential to create a platform for involving community stakeholders in the process of better understanding the system behaviors and designing solutions to manage policy changes by unbiased model-based insights.

In terms of limitations, to capture a clearer picture of the system, we need to understand how permanent housing services are being utilized to transition individuals from the “housing insecure” state to the “housed” state for each community. The model can then be modified based on these findings as the process may differ from one locality to another depending on the available services and processes developed by local agencies. Additionally, the model boundaries can be expanded beyond a specific region to capture relocations across county and state boundaries. Also, this study does not account for policy resistance and push-back from different subsystems and agencies. Based on the underlying structure developed in this study, future work can focus on identifying and obtaining a better understanding of the sources of policy resistance for different policy implications to develop more effective and sustainable solutions. Another limitation of this work is that the developed system dynamics model collectively analyzes the improvement in preventative initiatives and their impact and does not differentiate between various prevention programs and their extent. Moreover, the model developed in this study is a high-level model aimed to provide insights from a systems perspective into the effectiveness of the solutions to homelessness. However, this imposes a trade-off; although studying policy implications in smaller and limited-scope models is prudent, it may lead to underestimating less evident system structures in large complex problems. To address this challenge, once we fully understand the results provided by a small model, a more detailed model can be developed to further analyze undelineated policy implications. Lastly, we would like to emphasize that to precisely portray the system elements that play a decisive role in alleviating homelessness, the model construction process should include several model extension and simplification phases.

## 7. Conclusions

With system dynamics models, we are able to represent the feedback process, time delays, and nonlinearities to determine the system’s behavior and patterns subject to new policy changes, which could help better understand how different policies and strategies in the real system would affect the at-risk population or individuals experiencing homelessness. This study is the first step in modeling and studying the impact of various high-level policy implications and their combined effect on homelessness. Further work is needed to expand the model and study the impact of various prevention policies and initiatives related to the contributing factors to homelessness.

## Figures and Tables

**Table 1:** Scenario analysis for system performance by applying the “Time to achieve 30% drop in homeless count” measure (in this table, Y stands for “years”). Homeless count includes the housing insecure population in the shelters and the ones not utilizing the temporary housing units.

		P2								
		0%			5%			10%		
		P3			P3			P3		
		0%	5%	10%	0%	5%	10%	0%	5%	10%
**P1**	5%	never	>30 Y	28 Y	never	>30 Y	25 Y	never	>30 Y	24 Y
	15%	never	23 Y	14 Y	>30 Y	16 Y	11 Y	>30 Y	15 Y	10 Y
	25%	>30 Y	15 Y	11 Y	20 Y	10 Y	8 Y	17 Y	9 Y	8 Y

## Data Availability

Not applicable.
